# The Influence of Running and Dancing on the Occurrence and Progression of Premenstrual Disorders

**DOI:** 10.3390/ijerph18157946

**Published:** 2021-07-27

**Authors:** Joanna Witkoś, Magdalena Hartman-Petrycka

**Affiliations:** 1 Faculty of Medicine and Health Science, Andrzej Frycz Modrzewski Krakow University, G. Herlinga-Grudzińskiego Street 1, 30-705 Kraków, Poland; 2 Department of Basic Biomedical Science, Faculty of Pharmaceutical Sciences in Sosnowiec, Medical University of Silesia, Katowice, Poland, Kasztanowa Street 3, 41-200 Sosnowiec, Poland; mhartman@sum.edu.pl

**Keywords:** premenstrual disorders, physical activity, run, dance

## Abstract

*Background*: The aim of the study was to assess the influence of both physical activity, such as running and dancing, and the personal characteristics of the studied women on the occurrence and progression of premenstrual disorder (PMD). *Methods*: We surveyed 414 women aged 22–48 who were experiencing the menstrual cycle but not using hormonal contraception. There were two physically active groups, runners (*N* = 215) and Argentine tango dancers (*N* = 94), and there was one group not undertaking any physical activity—the control group (*N* = 104). The research was conducted using the researchers’ own questionnaire. *Results*: The number of days of PMD symptoms in the tango vs. runner vs. control groups are as follows: pre-bleeding (mean: 4.14 vs. 4.86 vs. 4.85; *p* = 0.024), after the onset of bleeding (mean: 1.76 vs. 2.39 vs. 2.16; *p* = 0.001), and in total (mean: 5.94 vs. 7.25 vs. 7.01; *p* < 0.001). The regression analysis results without grouping results are as follows: the number of days of symptoms before bleeding and menarche (B: −0.16; 95% CIs: from −0.29 to −0.04; *p* = 0.011), the total duration of symptoms and menarche (B: −0.17; 95% CIs: from −0.32 to −0.01; *p* = 0.036), lower abdominal pain and age (B: −0.05; 95% CIs: 0.92–0.98; *p* = 0.002), diarrhoea (B: −0.08; 95% CIs: 0.88–0.97; *p* < 0.001), tearfulness, depressive states and age (B: −0.06; 95% CIs: 0.91–0.97; *p* < 0.001), skin problems and age (B: −0.05; 95% CIs: 0.92–0.98; *p* = 0.004), joint pain and age (B: −0.09; 95% CIs: 0.86–0.96; *p* = 0.001), pain in the lumbar spine (B: −0.06, 95% CIs: 0.91–0.98; *p* = 0.001), water retention and BMI (B: 0.09; 95% CIs: 0.92–0.98; *p* = 0.007), and water retention and menarche (B: −0.19; 95% CIs: 0.73–0.94; *p* = 0.003). *Information*: generally there is one regression model, we have several here, we have a bit the description. *Conclusions*: Physical activity such as dancing (tango) shortens the duration of PMD symptoms but does not completely eliminate them. Running does not have as beneficial an effect on symptom relief as dancing. Current age, age when menstruation began (menarche), and BMI were revealed to be important factors influencing the symptoms of premenstrual disorders.

## 1. Introduction

A woman’s monthly cycle is a complex physiological process and the most important indication that the reproductive system is functioning. Hormonal changes occurring during the menstrual cycle are regulated by the hypothalamic–pituitary–gonadal axis (HPG axis) and are designed to induce ovulation and changes in the uterine mucosa to enable a woman to become pregnant. The monthly cycle, which usually lasts about 28 days, is divided into two phases: follicular (days 1–14), which begins on the first day of bleeding and ends with ovulation, and luteal (days 15–28), which lasts until the next menstrual period [1].

Most women of childbearing age experience a range of physiological, psycho-emotional, and behavioural changes that occur in conjunction with the menstrual cycle. These symptoms usually occur in the luteal phase and are generally referred to as premenstrual disorders (PMD) [2,3], including premenstrual syndrome (PMS) [4,5,6] and premenstrual dysphoric disorder (PMDD) [7,8,9]. The American College of Obstetricians and Gynaecologists (ACOG) [10] defines PMS as a clinical condition that includes somatic and emotional symptoms unrelated to any organic disease, appearing within five-to-seven days preceding menstruation in any of the three previous menstrual cycles and disappearing within two-to-four days after the start of bleeding. Typically, PMS symptoms are not reported and do not occur until at least the 13th day of the next cycle [10]. On the other hand, the American Psychiatric Association (APA) has established the criteria for diagnosing a much more severe and more serious variant of PMS, i.e., PMDD [11]. It is characterised by debilitating somatic and behavioural symptoms, including a dysphoric mood with a possible intensity up to a major depressive episode (MDE) that significantly affects the quality of life of the women affected by it or even completely prevents a woman from functioning on an everyday basis [11]. PMDD is a relatively newly diagnosed psychiatric disorder that affects fewer women than PMS. PMDD is defined as occurring when a woman suffers from at least five different psychological premenstrual symptoms, such as depression, anxiety, sadness, or emotional lability. The prevalence of PMDD, as well as PMS, in women emphasises the interrelationship of these diseases with depression, and this is supported by an increased frequency of comorbidity of major depressive disorder (MDD) in women experiencing PMS and PMDD [11]. Research results have shown a relationship between endogenous depression and the depressive episodes occurring in the luteal phase of the cycles of women with PMS. However, despite the segregation of PMS and PMDD, it should be borne in mind that the transition between them is quite smooth and common causes are sought for both entities [12]. 

PMD is one of the most common female health problems around the world and one of the most widespread cyclical psychosomatic disorders that significantly interferes with the daily functioning and rhythm of women’s life. Furthermore, it increases stress sensitivity. It has been reported that, before menstruation, women experience an increased sensitivity to environmental stress. Women also report that heightened sensory perception is more severe for them, and this makes everyday chores more onerous. This has led to the suggestion that the mere appearance of PMD symptoms causes anxiety and is a stressor for a woman [13]. 

Monthly differences in hormone levels lower the quality of life of women of reproductive age, and they disturb personal (partnership and family) and social relationships including professional work (loss of productivity and absenteeism), which also has economic consequences. PMD is therefore classified as a psychosocial problem [14]. The scale of the impact of the negative changes preceding menstruation shows that this is a major health problem for women and, in the health field, an important topic for research, which includes especially focusing on ways and options to alleviate and treat PMD. 

The actual prevalence of PMD is difficult to establish due to the many factors that can determine it, including differences in access to medical care, diagnostic criteria, methods used to identify and classify cases, symptom diversity, self-medication, underlying diseases, local culture, failure to report such symptoms to the doctor because the woman considers them an inevitable effect of hormonal changes occurring before menstruation, and a lack of knowledge that PMD can be treated. Even so, epidemiological studies estimate that the incidence of PMS symptoms is 80–90%, while 3–8% of women suffer from PMDD [15]. It is likely, however, that the number of women suffering from PMDD is greater, as many women may “lack,” for example, just one of the symptoms that meet the arbitrary criteria of the five symptoms required to make a diagnosis of PMDD. According to data from the World Health Organisation (WHO), painful periods affect 1.7–97% of women [16]. It has been shown that most women report at least one symptom of PMD and that a woman is able to function normally at work and at home, while the percentage of women with more than one symptom ranges from 20% to 40% [16].

About 150 symptoms of PMD have been reported, but the most commonly described affective symptoms include depressed mood (depression), feelings of loneliness, sadness, tendency to cry, unhappiness, outbursts of anger, irritability, emotional lability, anxiety, difficulty concentrating, confusion, forgetfulness, anxiety, sleep disturbance, fatigue, and social withdrawal including from partner and family. Somatic symptoms include, but are not limited to, pain (abdominal, head, back, muscle, and joint pain), breast tenderness, flatulence, nausea, limb swelling, weight gain, constipation or diarrhoea, changes in appetite, general lack of energy, excessive sleepiness or insomnia, increased heart rate, and skin problems [2,3,4,5,6,7,8,9,17].

All the symptoms of PMD negatively affect the daily life of women of childbearing age. It is difficult to estimate which of the symptoms is particularly severe and, therefore, affects daily functioning the most, but it seems that it may be abdominal pain and cramping, which often radiate towards the groin, back, or thighs and are accompanied by other symptoms such as nausea and/or diarrhoea. They occur earlier than 12 months after the menarche and are most common in the first 8–72 h of bleeding. Pain is a complex sensory experience that affects mood, behaviour, and exclusion from daily life activities, and it can also modify thought patterns, thus leading to the activation of various brain regions during cognitive tasks. Menstrual pain or dysmenorrhea can be classified as primary and secondary. Primary dysmenorrhea results from excessive, pathological contractions of the uterus without any other pathological changes in the pelvic area. Secondary dysmenorrhea is associated with the incidence of changes, such as endometriosis, chronic pelvic inflammation, uterine fibroids, endometrial polyps, and cervical stenosis, as well as anatomical and functional abnormalities of the generative organs [2,3,4,5,6,7,8,9,17]. 

The pathophysiology and exact pathogenic mechanisms of PMS and PMDD remain unknown. However, it can certainly be said that they are complex and multifactorial, as it is not possible to identify one main factor contributing to the formation of PMD, but rather it is a combination of several possible components. As the symptoms of PMD are closely connected with the menstrual cycle and only affect women of childbearing age, it has been suggested that sex hormones play a causal role in the pathogenesis of this syndrome. Women with PMS and PMDD appear to have an abnormal response to normal changes and gonadal steroid hormone levels over the menstrual cycle [2,3,4,5,6,7,8,9]. 

The increased neurobiological sensitivity of the central nervous system to changes, differences, and fluctuations in the level of sex hormones during the menstrual cycle contributes to the occurrence of PMD. Symptoms occur in women of childbearing age, and they do not occur before the menarche nor after the menopause, which also confirms that it is the fluctuations in hormones that may be of key importance to the onset of PMD symptoms [18]. Oestrogen and progesterone fluctuations, neuroendocrine disorders, and prostaglandin synthesis play a role in the biological aetiology of PMD. Oestrogen affects neurotransmitters such as serotonin, noradrenaline, gamma-aminobutyric acid, dopamine, and acetylcholine that regulate mood, behaviour, and cognition [18]. Therefore, the decline in oestrogen levels in the luteal phase plays a role in the development of PMD symptoms. It should also be remembered that the above hormones, apart from their reproductive function, have a huge impact on many tissues of the body, including skeletal muscles, heart, bones, connective tissue, and the central and peripheral nervous system [2,3,4,5,6,7,8,9]. 

The period of sexual maturation and the related role of sex hormones play important roles in the neurodevelopment of young women [19]. The increase in and cyclical nature of hormones during puberty have been shown to modulate the sensitivity of the neuroendocrine system, which may lead to symptoms of PMD [13]. Steiner et al. [20] showed that most adolescents reporting severe PMS and PMDD confirmed that their symptoms started with menarche. However, there have been no conclusive studies on the timing of menarche in girls (early/late) as a predictor of PMD and PMDD. For example, research [21] results showed that women with PMD were younger when they had their first menstrual period than those without symptoms. The authors’ findings [21,22,23,24] showed that the timing of puberty development, in particular the timing of menarche, is inversely related to the risk of PMD symptoms in adulthood. It could be speculated that those who experience an early menarche have more ovulatory cycles and, consequently, more exposure to hormone fluctuations at a certain age, so they are more susceptible to PMD than those with a later menarche. It should also be noted that the relationship between menarche and PMD is also associated with the occurrence of depression, which is in turn positively associated with an early age of menarche. In addition, it is known that rigorous physical activity can be associated with late menarche and, therefore, with a reduced risk of PMD [25,26]. However, little is known about what life factors of pre-menarche life may play significant roles in the development of PMD.

Some studies [27] of the causes of PMD have found more serotonin abnormalities in the luteal phase in women with PMS compared to women without such symptoms. The neurotransmitter serotonin is responsible for creating and controlling many physiological, behavioural, and emotional responses. Because the symptoms of PMD are similar to those where there is a reduced level of the neurotransmitter serotonin, it is believed that serotonin may play a role in the etiopathogenesis of this condition. Additionally, it has been noted that women with serotonin deficiency have an increased sensitivity to progesterone, which is also considered a factor responsible for this type of disorder. Other possible causes of PMD include genetic factors, which has been confirmed by many studies that have shown that up to 57% of surveyed women had a familial trait, i.e., with their mother and siblings. Studies with twins also suggest the presence of a genetic component in PMD [27].

Further causes are the impairment of the renin–angiotensin–aldosterone pathway, hyperprolactinaemia, an increased sensitivity to the effects of prolactin, insulin resistance and changes in glucose metabolism, sensitivity to endogenous hormones, a malfunction of the hypothalamic–pituitary–adrenal axis, nutritional deficiencies, fluid and electrolyte imbalance (fluid retention in the body), and thyroid disorders. It has also been shown that the increased secretion and/or imbalance of prostaglandins and other inflammatory factors can stimulate excessive uterine contractility, leading to decreased blood flow through this organ and, subsequently a low level of oxygen, as well as symptoms such as nausea and headache. Among the possible causes, the dysregulation of the main metabolite of progesterone, allopregnanolone, and its effect on the neurotransmitters such as GABAergic (GABAA—gamma-aminobutyric acid) and serotonergic, as well as opioids and catecholamines, are also being considered [2,3,4,5,6,7,8,9].

Undoubtedly, a woman’s lifestyle and everyday environment, the level of perceived stress, mental state, physical activity, diet, and the habits that are unfavourable to health have strong influence on the occurrence of PMD symptoms [28,29,30]. Bertone-Johnson et al. [31] pointed out that factors such as pre-existing serious mood disorders and a history of sexual, emotional, and physical abuse; exposure to domestic violence and stress levels such as a stressful job; or stress in the home or school environment may also contribute to the pathogenesis of PMD. Studies [20] have shown that women with PMS have constant emotional disturbances and an increased reactivity to stress, independent of the menstrual cycle, and this may indicate abnormal neural and physiological reactivity under stressful conditions [32,33,34,35]. Almost 30% of women who have had dysmenorrhea during each cycle have reported experiencing stress on a daily basis [36]. Stress changes mood by reducing the secretion of beta-endorphin in the brain and cortisol in the adrenal cortex, and this gives rise to psychological symptoms and mood changes. Some studies have found that the onset and progression of PMS are directly related to stress. It has also been shown that the occurrence of PMDD is associated with a greater sensitivity to stress in women suffering from this syndrome [32,33,34,35]. Olson et al. [33] confirmed that stress can worsen PMS symptoms. In addition, women who smoke, trying to reduce their anxiety with the use of a stimulant such as tobacco, create the conditions for PMS symptoms [37]. PMS has been shown to be more common in women who smoke than non-smokers. Growing scientific evidence shows that psychosocial factors are associated with the development and progression of premenstrual symptoms, as well as that psychological interventions are effective in reducing the symptoms of PMD. This suggests that factors should be considered that make it easier for women to cope with the negative feelings that occur before menstruation. “Coping” is understood to be a change in the way of thinking, feeling, and behaving when a situation arises that is considered difficult and stressful [38].

As the aetiology of PMD is still unclear, identifying modifiable risk factors is important in improving the quality of life of many women of childbearing age worldwide [34]. Therefore, the aim of this study was to assess the impact of regular physical activity such as running and tango dancing on women of reproductive age, as well as to assess the occurrence of PMD symptoms in the groups. The relationships between the role of physical activity, BMI level, age of women, age of menarche, and symptoms of PMD were examined in the studied women.

## 2. Materials and Methods

### 2.1. Participants

The study involved 414 women aged 22–48 years, all of whom were menstruating naturally and did not use hormonal contraception. Two groups of physically active women, runners (Group B; *N* = 215) and tango dancers (Group T; *N* = 94), and one group of sedentary women who declared a lack of any regular physical activity, the control group (Group K; *N* = 104), participated in the study. The characteristics of the studied women, including their monthly cycles and training parameters, are presented in Table 1 and Table 2. 

### 2.2. Survey

The research was carried out using a proprietary survey including questions about the demographic data of the women, such as age, height, weight, and the progress of their menstrual cycle. Next, they were asked about the symptoms occurring before menstrual bleeding and accompanying menstrual bleeding. Additionally, in the physically active groups, there was a question about how long the women had been engaged in the given type of physical activity and how much time a week they devoted to this activity (Appendix A). The questionnaires were distributed online using Google forms. The questionnaire for runners and dancers was posted on thematic social networks devoted to the given physical activity. Surveys in the control group were obtained from a group of female students from one of the universities in Krakow and from their family and friends. The questions included in the survey were based on the previous research connected with this topic and cited in this publication in the Section 4. 

### 2.3. Statistical Analysis

Statistical analysis was performed using the SPSS 21 software program, Version 27.0, IBM Corp., Armonk, NY, USA. The significance of differences in the occurrence of symptoms and the course of PMD between the runners, dancers, and control groups was calculated using the analysis of variance and the Games–Howell post hoc test for numerical variables, as well as the chi square test for dichotomous and qualitative variables.

Multiple linear regression analysis was performed to evaluate the effect of predictors such as group, age, BMI, and age of first menstruation (menarche) on the duration of menstrual-related PMD symptoms. To assess the influence of predictors such as group, age, BMI, and age of the first menstruation (menarche) on the occurrence of PMD symptoms and various symptoms of PMD itself, multiple logistic regression analysis was performed. The results were considered statistically significant when *p* < 0.05.

## 3. Results

The women participating in the study, belonging to the three study groups, did not significantly differ in age, height, or age of the first menstruation, but they differed in body weight, BMI, number of days of PMD symptoms occurring before and after menstruation, and the total number of days their PMD symptoms lasted (Table 1). Women from the control group had the highest body weight and the highest BMI—significantly higher than the dancers and runners. The dancers, on the other hand, were significantly slimmer than not only the control group but also the runners. The fewest number of days PMD symptoms, which occurred before menstruation, was in the tango group, and this was significantly fewer than in the control and runners groups. These symptoms were also resolved in the tango group significantly faster after the onset of bleeding compared to the runners group. The total duration of PMD symptoms also turned out to be the shortest in the tango group—significantly shorter than in the runners and control groups.

**Table 1:** Characteristics of the studied women, the year of their menarche, and the duration of PMD symptoms associated with menstruation in the runners (B; *N* = 215), tango dancers (T; *N* = 94), and control (K; *N* = 104) groups.

	Median			Mean			Standard Deviation			Minimum			Maximum			Statistics		Groups That Differ Significantly
Group	B	T	K	B	T	K	B	T	K	B	T	K	B	T	K	F	*p*	
Age [years]	35.0	37.0	36.0	34.7	35.7	35.2	6.2	5.5	6.7	22.0	22.0	22.0	48.0	45.0	48.0	0.88	0.416	
Height [cm]	165.0	165.0	165.0	166.2	165.4	166.1	5.9	5.7	7.1	152.0	153.0	148.0	184.0	183.0	185.0	0.60	0.548	
Body weight [kg]	60.0	58.0	68.0	61.6	58.5	69.7	9.5	7.1	14.8	45.0	45.0	44.0	97.0	85.0	120.0	30.83	<0.001	BT, BK, TK
BMI [kg/m^2^]	21.8	21.0	24.2	22.3	21.4	25.2	3.0	2.4	4.7	17.1	18.0	17.2	34.4	31.6	37.5	35.83	<0.001	BT, BK, TK
Age of first menstruation (Menarche)	13.0	13.0	13.0	13.1	12.7	13.0	1.7	1.7	1.6	9.0	9.0	10.0	19.0	16.0	18.0	2.32	0.100	
The number of days of PMD before menstruation	5.0	4.0	5.0	4.9	4.1	4.9	1.7	2.1	2.6	1.0	1.0	1.0	8.0	7.0	14.0	3.78	0.024	BT, TK
The number of days of PMD from the first day of menstruation	2.0	2.0	1.0	2.4	1.8	2.2	1.4	0.9	1.4	1.0	1.0	1.0	7.0	4.0	5.0	6.79	0.001	BT
Total number of days of PMD	8.0	6.0	6.5	7.3	5.9	7.0	2.3	2.3	3.0	2.0	2.0	2.0	14.0	10.0	19.0	7.79	<0.001	BT, TK

In the physically active groups, the dancers had been dancing tango for 5.25 years for 6 h a week, while the runners had been training for 4 years for 3–4 h a week, covering distances from 5 to 16 km (Table 2).

**Table 2:** Training characteristics of the tango dancing women (*N* = 94) and the runners (*N* = 215).

Group	Training Characteristics	Median	Mean	Standard Deviation	Minimum	Maximum
Tango	How many years have you been dancing the tango? [years]	5.25	6.63	4.66	0.50	23.00
	How much time do you spend dancing tango per week?	6.00	7.65	6.32	0.00	30.00
Runners	How many years have you been running? [years]	4.00	5.41	4.42	1.00	30.00
	What are the minimum distances you run? [km]	5.00	6.79	3.69	1.50	40.00
	What are the maximum distances you run? [km]	16.00	19.83	12.14	5.00	80.00
	What is the minimum number of times week you run?	3.00	3.27	1.19	1.00	7.00
	What is the maximum number of times a week you run?	4.00	3.99	1.21	1.00	7.00
	What is the minimum number of hours of one run? [h]	1.00	1.01	0.34	0.50	2.00
	What is the maximum number of hours of on run? [h]	1.30	1.53	0.78	0.50	5.00

The percentage of people suffering from symptoms related to PMD in the runners, tango, and control groups did not differ in a statistically significant manner and fluctuated around 90% (Table 3). There was also no significant difference in the frequency of individual PMD symptoms. However, there was a tendency (*p* = 0.057) for a higher incidence of nausea and vomiting in the dancers group, as well as a tendency (*p* = 0.061) for less abdominal pain in this group. 

Multiple linear regression analysis confirmed a statistically significant effect of belonging to a specific study group on the duration of PMD symptoms, both before and after the onset of bleeding, and on the overall duration of these symptoms (Table 4). Multiple intergroup comparisons showed that the symptoms of PMD were the shortest in the group of dancers (Table 5). In addition, linear regression analysis showed that the negative predictor for the duration of PMD symptoms before the onset of bleeding and for the cumulative duration of menstrual problems was the year in which menstruation first began (Table 4).

During regression analysis, it was shown that none of the predictors significantly influenced the occurrence of PMD symptoms (Table 6). Regression analysis also did not show a significant influence on the occurrence of a particular symptom of PMD with regard to the women belonging to a particular study group. The analysis of predictors for individual symptoms of PMD showed that the woman’s age had a significant influence on the occurrence of numerous symptoms. Younger women were more likely to experience lower abdominal pain, diarrhoea, tearfulness and depression, skin problems, joint pain, and pain in the lumbar spine. It was also observed that water retention is more common in women with a higher BMI and in women who had their first menstrual period at a younger age.

## 4. Discussion

The main objective of this study was to assess the impact of regular physical activity such as running and tango dancing on women of reproductive age, as well as the relationship between physical activity and symptoms of PMD.

The authors of the manuscript arbitrarily chose the two above-mentioned forms of physical activity because they appear to be quite popular with women. Running is probably the cheapest form of physical recreation undertaken by women of all ages that, for all intents and purposes, does not require any financial resources. Dancing the tango, on the other hand, requires more financial resources and is aimed at a slightly more sophisticated audience. Its bygone popularity, however, is seeing a resurgence, which is reflected in the number of new schools offering this form of dance. The number of women who choose this type of physical activity in order to satisfy their different personal needs is also definitely increasing [39]. Tango, compared to other forms of ballroom dancing, is characterised by simple steps that are easy and quick to learn. Moreover, what may be important for many women is the fact that this dance does not require a particular level of physical fitness or performance, nor a slender figure, from the dancer.

By choosing the two above-mentioned forms of physical activity, which appear to be quite distinct from each other in their form and style, the manuscript authors wanted to reach two decidedly different groups of women who prefer a diverse style and type of physical activity in order to research the influence of both dynamic and slightly calmer forms of movement on PMD symptoms.

This study found that although the percentage of women who suffered from PMD symptoms did not differ significantly in any of the studied groups, the tango dancers reported a shorter duration of symptoms, both before and after the onset of bleeding, compared to the women from other two groups (runners and control). Therefore, the type and dynamics of physical activity undertaken by women may be considered regarding the occurrence of PMD symptoms. Running is a rather dynamic and strenuous activity, while dancing, especially tango, is actually a “walk with a partner.” Perhaps such a calm and “undemanding” form of exercise, affecting the muscles of the pelvic and abdominal area, relaxes and oxygenates them so significantly that it is a better form of relieving PMD symptoms than running. 

Many of the world’s leading societies suggest physical activity as a first-line treatment in relieving PMD symptoms. This advice is given by the National Institute for Health and Care Excellence (NICE), the Royal College of Obstetricians and Gynaecologists (RCOG), as well as the American College of Sports Medicine (ACSM), and the International Society for Premenstrual Disorders (ISPMD) [40]. Physical activity is a recommended and appropriate form of reducing the severity of PMD symptoms. However, there is still too little scientific evidence as to what form of activity is the most effective, which activity should be recommended, and how many times a week it should be undertaken. The fact that physical activity may also exacerbate premenstrual symptoms has not been clearly ruled out, so further follow-up research in this area is needed. 

Vishnupriya et al. [41] showed that PMS symptoms were significantly reduced when women undertook moderate-intensity aerobic exercise. Maged et al. [42] studied women who swam in a pool and found that swimming has a beneficial effect on most of the physical and psychological symptoms of PMS. There was a significant difference between the positive effect on the reduction of most psycho-emotional and physical symptoms, such as headache, fatigue and swelling, and breast tenderness, between the test group undertaking physical activity and the control group. In a study by Azhary et al. [43], a significant reduction in pain in the abdomen and the lumbosacral region was noted, as was reduced swelling in women undertaking physical activity. Vaziri et al. [44] compared the effects of aerobic and stretching exercises and found that both forms of exercise help relieve PMD symptoms. Mohebbi-Dehnavi et al. [45] showed that eight weeks of aerobic exercise was associated with a significant decrease in PMS symptoms, though only the physical ones such as headache, nausea, diarrhoea, and swelling. Aerobic exercise reduces renin levels, increases concentrations of oestrogen and progesterone, and lowers concentrations of serum aldosterone levels, all of which reduce the above-mentioned symptoms [46]. The authors attributed the difference in the results of this study and other studies to differences in exercise duration and intensity, eating habits, and the environment in which the surveyed women were living. Lustyk et al. [47] showed that women who only occasionally engage in physical activity have more severe PMS symptoms than those who regularly exercise. Kitamura et al. [48] proposed a set of exercises that were able to significantly reduce the severity of pain during menstruation. They also had an impact on the duration of menstruation and the amount of blood loss. Research by Su-Ying Tsai [49] showed a link between exercise and PMS and revealed that a regular exercise habit can reduce some physical and psychological PMS symptoms. This study showed that women with PMS participating in short-term yoga exercises during the luteal phase felt better and had improved attention. Another study found that mean PMS scores and symptoms decreased after eight weeks of aerobic training in an experimental group and suggested that eight weeks of aerobic exercise was effective in reducing PMS symptoms and could be used as a treatment [49,50]. Yoga induced an increase in alpha wave production, which is closely related to slower abdominal breathing. It has a positive effect on brain wave activity, and brain alpha waves are associated with states of calmness, relaxation, creativity, mood elevation, and the release of serotonin. This increase in brain alpha waves suggests that participants felt more relaxed after yoga. Growing evidence shows that yoga benefits physical and mental health by alleviating the regulation of the hypothalamic–pituitary–adrenal axis and the sympathetic nervous system. Yoga has become an increasingly popular form of physical recreation among people with pain [49,50]. 

In contrast, Kritz-Silverstein et al. [30], and Sadler et al. [51] showed no relationship between PMD and aerobic exercise and sport in general. Aimee et al. [52] also found that there no significant relationship between exercise and PMS. It should be noted that not all of the above-mentioned the authors considered other factors that could influence PMD, such as BMI, which was positively correlated with with water retention in our study. Other authors recommend physical activity and have reported that aerobic exercise reduces PMD symptoms in women who exercise regularly. On the other hand, Taheri et al. [53] found that the risk of dysmenorrhea was adversely associated with regular exercise and healthy exercise in women. Similar results were described by Deuster et al. [54], Rasheed et al. [55], and David et al. [56], whose studies have shown a negative impact of exercise on PMD symptoms and higher levels of premenstrual symptoms among women who exercise more compared to those who exercise less or not at all. 

Physical activity has well-known advantages, such as improving general physical fitness, weight loss and BMI normalisation, and increasing the release of neurotransmitters, including endorphins, dopamine, and endogenous opiate peptides [57], which not only help to improve the mood but also affect the proper synthesis of progesterone and oestrogens and contribute to the production of endogenous anti-inflammatory substances [58]. Exercise reduces the levels of estradiol and other steroid hormones, improving the transport of oxygen in the muscles and raising the level of cortisol [59]. Sabaei et al. [60] showed the effectiveness of exercise in reducing blood leptin levels by 30–34%, which also helped reduce PMS behavioural symptoms because high levels of circulating leptin are associated with the psychological symptoms of PMS. 

Exercise is also an opportunity to socialise and thus reduce social isolation and minimise stress and anxiety, leading to improved mental well-being and acting as a ‘distraction’ from intrusive thoughts, which improves mood and prevents depression [58,59]. Since stress and anxiety are proportionally related to PMS symptoms and menstrual pain, reducing them can significantly lessen menstrual pain and PMS symptoms. Because it stimulates the sympathetic part of the autonomic nervous system, stress leads to an increase in menstrual pain by increasing the intensity of uterine contraction. Being physically active relieves stress levels, which are correlated with menstrual pain and other symptoms of PMD [60]. All of the above-mentioned benefits of physical activity may contribute to the at-least partial relief of PMD symptoms, so this method of alleviating these unpleasant symptoms is recommended as an alternative to pharmacological treatment. 

Cross-sectional studies have shown that PMD is as common in teenage girls as it is in adult women [20]. The findings of many authors [20,61,62], however, have shown that the time of puberty, especially menarche, is inversely related to the risk of PMD symptoms in adulthood. The results in this study regarding the time of menarche and the symptoms of PMD reported by the studied women are in line with the results from the literature. Symptoms of PMD before menstrual bleeding and the total duration of these symptoms have been shown to be longer in women who developed menarche at a younger age. It has also been shown that many of the symptoms of PMD decrease with age. Water retention was also shown to be more frequent in women who had their first period at a younger age. This study has shown that age is an important factor in the occurrence of some PMD symptoms, both somatic and affective. Younger women were found to more often suffer from lower abdominal pain, diarrhoea, skin problems, joint pain, pain in the lumbar region of the spine, and tearfulness and depression. This was declared more often than in the other groups. 

Research into the factors that may predispose women to pain during menstruation have shown that one of the causes may be BMI [63,64]. A high BMI, indicative of obesity, has been positively correlated with PMS symptoms. It has been observed that obese women who have a significant accumulation of visceral fat often struggle with painful menstruation. This research did not show that a higher BMI significantly influenced the symptoms appearing during the menstrual cycle. It only showed that water retention, one of the symptoms of PMD, was more common in women with a higher BMI. 

Certainly, dietary and nutritional modifications are essential for overall better health, including avoiding high-calorie and highly processed foods, fast food, and foods high in salt, sugar, and fat but low in nutrients. Studies by other authors have shown that dysmenorrhea is much more common in girls who often eat fast food [63]. Houghton et al. [64] showed, however, that the consumption of carbohydrates and fibre reported by women did not contribute to the risk of PMS. In research by Mohebbi et al. [26], it was confirmed that in groups of women with and without PMS, there was a difference in both eating habits, which comprise an element of lifestyle, as well as perceived stress and a tendency to unhealthy behaviours such as drinking alcohol or smoking. The authors also noted that the average consumption of a variety of foods significantly differed between the two groups. In several studies [26,63,64], the impact of eating habits and their relationship with PMS were assessed, and it was found, for example, that increasing the amount of carbohydrates in the diet may improve mood and reduce the severity of the physical symptoms of PMS. A study by Cross et al. showed that women with PMS consumed more fat, carbohydrates, and simple sugars and received significantly less protein before menstruation compared to women without PMS. In the blood serum of women with symptoms of PMD, changes/deficiencies resulting from unhealthy eating habits were also found in the levels of magnesium and calcium and vitamins B2 (riboflavin), B3 (niacine) B6 (pirydoxine), and B12 (cobalamin). These B vitamins are essential for the metabolism of dopamine and serotonin. There was also a lack of folic acid and essential fatty acids [26,63,64]. 

Despite the high prevalence of PMD symptoms among women of childbearing age, there are relatively few treatment options for the condition. Estimates show that 30–40% of women require treatment. Treatment methods mainly include lifestyle changes, cognitive and behavioural therapies using techniques that help reduce stress (such as massage, reflexotherapy, and yoga), reducing or stopping smoking, and the education of women about PMD. Furthermore, physical aerobic activity and pharmacological treatment, mainly focused on the use of selective serotonin reuptake inhibitors (SSRIs) and contraceptives, are recommended [7,65,66,67]. The above discussion shows that the main goal of PMD treatment is to relieve symptoms and reduce their impact on a woman’s daily activities.

## 5. Conclusions

Despite the fact that there were no differences in the percentage of women in the dancers, runners, and control groups who reported PMD symptoms, there was a reduction in the duration of the maladies involved in PMS in the group of tango-dancing women, both before and after the commencement of bleeding.

The age of the women, the time of the first menstruation, and BMI were shown to be important factors affecting PMD. Younger women experienced lower abdominal pain, diarrhoea, tearfulness and depression, skin problems, joint pain, and pain in the lumbar region of the spine more often. Water retention was more common in women who had their first menstrual period at a younger age and in women with a higher BMI. Symptoms of PMD before the commencement of bleeding and the total duration of symptoms were longer in women who experienced menarche at a younger age. 

Physical activity in the form of tango dancing is beneficial. It shortens the duration of PMD symptoms, but it does not completely eliminate them. Running does not have as significant and beneficial effect as dancing on the possible relief of PMD symptoms. Significant factors influencing PMD are age, time of first menstruation, and BMI.

## Figures and Tables

**Table 3:** Percentage of women in the runners (*N* = 215), tango (*N* = 94), and control (*N* = 104) groups experiencing various symptoms of PMD and pain at different times of menstruation and the duration of their menstrual cycle.

		Runners		Tango		Control		chi^2^	*p*
		N	%	N	%	N	%		
Incidence of PMD	Yes	195	90.70	82	87.23	96	92.31	1.53	0.466
PMD symptoms	Headache and dizziness	46	21.40	18	19.15	28	26.92	1.92	0.382
	Pain in the lower abdomen	125	58.14	41	43.62	57	54.81	5.59	0.061
	Diarrhoea	33	15.35	14	14.89	22	21.15	1.99	0.371
	Nausea and vomiting	8	3.72	10	10.64	6	5.77	5.72	0.057
	Irritability	143	66.51	57	60.64	74	71.15	2.45	0.294
	Tearful and depressive states	82	38.14	45	47.87	36	34.62	3.96	0.138
	Breast tenderness	116	53.95	42	44.68	59	56.73	3.23	0.199
	Water retention in the body	95	44.19	38	40.43	51	49.04	1.51	0.471
	Skin problems	74	34.42	30	31.91	40	38.46	0.97	0.615
	Fatigue	74	34.42	35	37.23	40	38.46	0.57	0.753
	Joint pain	22	10.23	7	7.45	15	14.42	2.61	0.271
	Pain in the ‘loins’	86	40.00	32	34.04	42	40.38	1.14	0.567
Menstrual pain	Painless	55	25.58	21	22.34	16	15.38	6.63	0.157
	Pain from the beginning	149	69.3	69	73.4	78	75		
	Pain all the time	11	5.12	4	4.26	10	9.62		
Length of menstrual cycle	<25 days	25	11.63	11	11.7	4	3.88	8.58	0.072
	25–31 days	158	73.49	74	78.72	89	86.41		
	>31 days	32	14.88	9	9.57	10	9.71		

**Table 4:** Multiple linear regression analysis of the predictors: group, age, BMI, age of menarche, and duration of PMD symptoms associated with menstruation.

Dependent Variables	Significance of the Model	Predictors	*B*	*p*	*95% PU*	
The number of days of PMD before menstruation	*LR* = 14.92 *p* = 0.011	Group		0.007		
		Age	0.02	0.171	−0.01	0.06
		BMI	−0.01	0.808	−0.07	0.05
		Menarche	−0.16	0.011	−0.29	−0.04
The number of days of PMD from the first day of menstruation	*LR* = 14.77 *p* = 0.011	Group		0.002		
		Age	−0.01	0.314	−0.03	0.01
		BMI	−0.01	0.597	−0.05	0.03
		Menarche	0.01	0.958	−0.08	0.08
Total number of days of PMD	*LR* = 19.92 *p* = 0.001	Group		<0.001		
		Age	0.01	0.506	−0.03	0.06
		BMI	−0.02	0.600	−0.10	0.06
		Menarche	−0.17	0.036	−0.32	−0.01

*95% PU*: 95% confidence intervals for *B*.

**Table 5:** Estimated means and standard error of duration of PMS symptoms related to menstruation in the runners, dancers, and control groups.

Group	The Number of Days of Tension Symptoms before Menstruation		Number of Days of Tension Symptoms from the Beginning of Menstruation		Total Number of Days of Tension	
	Estimated Mean	Standard Error	Estimated Mean	Standard Error	Estimated Mean	Standard Error
Runners	4.89	0.15	2.38	0.01	7.27	0.18
Tango	4.04	0.24	1.76	0.15	5.83	0.29
Control	4.88	0.23	2.19	0.14	7.07	0.28

**Table 6:** Multiple logistic regression analysis of the predictors of the occurrence of PMS and individual symptoms of PMS. *LR*: likelihood ratio chi-square; *p*: significance level for LR; OR: odds ratio; *95% PU*: 95% confidence intervals for odds ratios.

Dependent Variables	Significance of the Model	Predictors	*B*	*p*	*OR*	*95% PU*	
Occurrence of PMS	*LR* = 6.22 *p* = 0.285	Group	-	0.704	-	-	-
		Age	−0.04	0.132	0.96	0.91	10.01
		BMI	0.06	0.308	10.06	0.95	10.19
		Menarche	−0.10	0.333	0.91	0.74	10.11
Headache and dizziness	*LR* = 4.78 *p* = 0.443	Group	-	0.731	-	-	-
		Age	−0.02	0.269	0.98	0.94	10.02
		BMI	0.04	0.201	10.04	0.98	10.12
		Menarche	0.01	0.982	10.00	0.87	10.16
Pain in lower abdomen	*LR* = 15.47 *p* = 0.009	Group	-	0.105	-	-	-
		Age	−0.05	0.002	0.95	0.92	0.98
		BMI	−0.01	0.723	0.99	0.93	10.05
		Menarche	0.03	0.596	10.03	0.92	10.17
Diarrhoea	*LR* = 16.08 *p* = 0.007	Group	-	0.598	-	-	-
		Age	−0.08	<0.001	0.92	0.88	0.97
		BMI	0.04	0.286	10.04	0.97	10.12
		Menarche	0.10	0.216	10.11	0.94	10.29
Nausea and vomiting	*LR* = 7.07 *p* = 0.216	Group	-	0.076	-	-	-
		Age	−0.04	0.242	0.96	0.89	10.03
		BMI	−0.03	0.667	0.97	0.85	10.11
		Menarche	−0.06	0.669	0.95	0.73	10.22
Irritability	*LR* = 9.25 *p* = 0.100	Group	-	0.307	-	-	-
		Age	−0.03	0.123	0.97	0.94	10.01
		BMI	0.01	0.926	10.00	0.94	10.06
		Menarche	−0.12	0.059	0.88	0.78	10.01
Tearfulness and a state of depression	*LR* = 22.49 *p* < 0.001	Group	-	0.134	-	-	-
		Age	−0.06	<0.001	0.94	0.91	0.97
		BMI	0.01	0.712	10.01	0.95	10.07
		Menarche	−0.11	0.080	0.89	0.79	10.01
Breast tenderness	*LR* = 5.39 *p* = 0.370	Group	-	0.140	-	-	-
		Age	−0.01	0.394	0.99	0.96	10.02
		BMI	−0.04	0.237	0.97	0.91	10.02
		Menarche	−0.01	0.921	0.99	0.88	10.12
Water retention	*LR* = 22.20 *p* < 0.001	Group		0.826	-	-	-
		Age	−0.02	0.360	0.99	0.95	10.02
		BMI	0.09	0.007	10.09	10.02	10.16
		Menarche	−0.19	0.003	0.83	0.73	0.94
Skin problems	*LR* = 11.93 *p* = 0.036	Group	-	0.566	-	-	-
		Age	−0.05	0.004	0.95	0.92	0.98
		BMI	−0.02	0.527	0.98	0.92	10.04
		Menarche	−0.07	0.269	0.93	0.82	10.06
Fatigue	*LR* = 0.73 *p* = 0.981	Group	-	0.826	-	-	-
		Age	−0.01	0.702	0.99	0.96	10.03
		BMI	0.01	0.733	10.01	0.95	10.07
		Menarche	−0.01	0.927	0.99	0.88	10.13
Joint pain	*LR* = 14.73 *p* = 0.012	Group	-	0.428	-	-	-
		Age	−0.09	0.001	0.91	0.86	0.96
		BMI	0.01	0.768	10.01	0.93	10.11
		Menarche	0.07	0.498	10.07	0.88	10.29
Pain in the lumbar region of the spine	*LR* = 13.74 *p* = 0.017	Group	-	0.812	-	-	-
		Age	−0.06	0.001	0.95	0.91	0.98
		BMI	0.04	0.152	10.05	0.98	10.11
		Menarche	0.02	0.754	10.02	0.90	10.15

## Data Availability

The datasets used and/or analysed during the current study are available from the corresponding author on reasonable request.

## Appendix A. The Author’s Own Survey

1. Year of birth
2. Height [cm]
3. Body weight [kg]
4. Are you menstruating? 1—YES, 2—NO
5. Are you currently using hormonal contraception? 1—YES, 2—NO
6. At what age did menstruation begin for you?
7. Are you periods painful? 1—Painless 2—Painful at the beginning 3—Painful during the whole time
8. How often do you menstruate? 1—Every 24 days, 2—25–31 days, 3—Over 31 days
9. Do you experience premenstrual syndrome (PMD) symptoms before your period? 1—YES, 2—NO
10. Do you experience any of the following symptoms before or during your period:
➢ Headache and dizziness 1—YES, 2—NO
➢ Pain in the lower abdomen 1—YES, 2—NO
➢ Diarrhoea 1—YES, 2—NO
➢ Nausea and vomiting 1—YES, 2—NO
➢ Irritability 1—YES, 2—NO
➢ Tearfulness and depressed state 1—YES, 2—NO
➢ Tenderness in breasts 1—YES, 2—NO
➢ Water retention in body 1—YES, 2—NO
➢ Skin problems 1—YES, 2—NO
➢ Fatigue 1—YES, 2—NO
➢ Joint pain 1—YES, 2—NO
➢ Pain in the lumbar spine 1—YES, 2—NO
11. How many days prior to bleeding do you experience PMD symptoms?
12. How many days after the commencement of menstrual bleeding do you experience symptoms of PMD?
**Only for the group of Argentinian Tango dancers**
13. How many years have you been dancing the tango?
14. How much time do you spend weekly dancing the tango [h]?
**Only for the group of runners**
13. How long have you been running for? [years]
14. What is the minimum distance you run? [km]
15. What is the maximum distance you run? [km]
16. What is the minimum number of times you run in a week?
17. What is the maximum number of times you run in a week?
18. What is the minimum number of hours for one run?
19. What is the maximum number of hours for one run?
